# Predictive value of acute atrial fibrillation for short-term mortality after ischemic stroke

**DOI:** 10.3389/fcvm.2026.1828038

**Published:** 2026-08-13

**Authors:** Guangchen Duan, Xiaoguang Zhu, Jiangshan Deng, Dongcheng Shi, Jiamei Jiang

**Affiliations:** 1 Emergency Internal Medicine, The Sixth People's Hospital Affiliated to Shanghai Jiao Tong University School of Medicine, Shanghai, China; 2 Department of Neurology, The Sixth People's Hospital Affiliated to Shanghai Jiao Tong University School of Medicine, Shanghai, China

**Keywords:** 30-day mortality, acute atrial fibrillation, acute ischemic stroke, ischemic stroke, mortality

## Abstract

**Background:**

Atrial fibrillation (AF) is an important risk factor for ischemic stroke. However, the prognostic impact of acute atrial fibrillation (AAF) at the onset of acute ischemic stroke (AIS) remains unclear.

**Methods:**

This retrospective study categorized 417 patients with AIS into the AAF (*n* = 72), other AF (*n* = 142), and non-AF (*n* = 203) groups. Multivariate logistic regression analysis of associations with 30-day all-cause mortality and severe early neurological deficit (7-day NIHSS ≥16).

**Results:**

The AAF group demonstrated significantly worse outcomes than the other AF and non-AF groups. In the multivariable analysis, AAF was identified as an independent risk predictor for severe 7-day neurological deficit [odds ratio (OR): 10.09; 95% CI: 3.87−27.36; *P* < 0.001] and all-cause mortality within 30 days (OR: 4.11; 95% CI: 2.28−7.43; *P* < 0.001).

**Conclusions:**

AAF at the onset of AIS is an independent risk predictor for early neurological deterioration and short-term mortality, establishing it as a crucial prognostic indicator that warrants vigilant management.

## 1 Introduction

Atrial fibrillation (AF) accounts for about 2%–4% of adults, and its incidence has gradually increased in recent years (1, 2). AF is the most common arrhythmia, and it is a key risk factor in the emergency room with acute ischemic stroke (3, 4). Approximately 15% of strokes are linked to AF, and ischemic strokes associated with AF tend to be more debilitating and have a higher mortality risk than other subtypes (5).

Acute atrial fibrillation (AAF) is defined by the American Heart Association as AF detected or manifested during an emergency department admission (6, 7). Patients with AAF during AIS presentation were distinguished from those with pre-existing AF, representing 25%–36.2% of patients after their first stroke (8). Patients with AAF, particularly following a cerebrovascular event, are at risk of recurrence, stroke, and poor outcomes (9). Consequently, recognizing the impact of AAF on AIS outcomes is key to therapeutic decision-making, including the timing of cardiac monitoring and anticoagulation to prevent stroke recurrence (10).

Although the emergency department manages most initial AAF presentations (11), the impact of these common episodes on AIS outcomes is poorly defined despite the critical nature of emergency department-based anticoagulation decisions for stroke prevention. This study aimed to assess the predictive value of AAF for early neurological deterioration and short-term mortality in AIS patients.

## 2 Materials and methods

### 2.1 Study protocol

**Figure 1:**
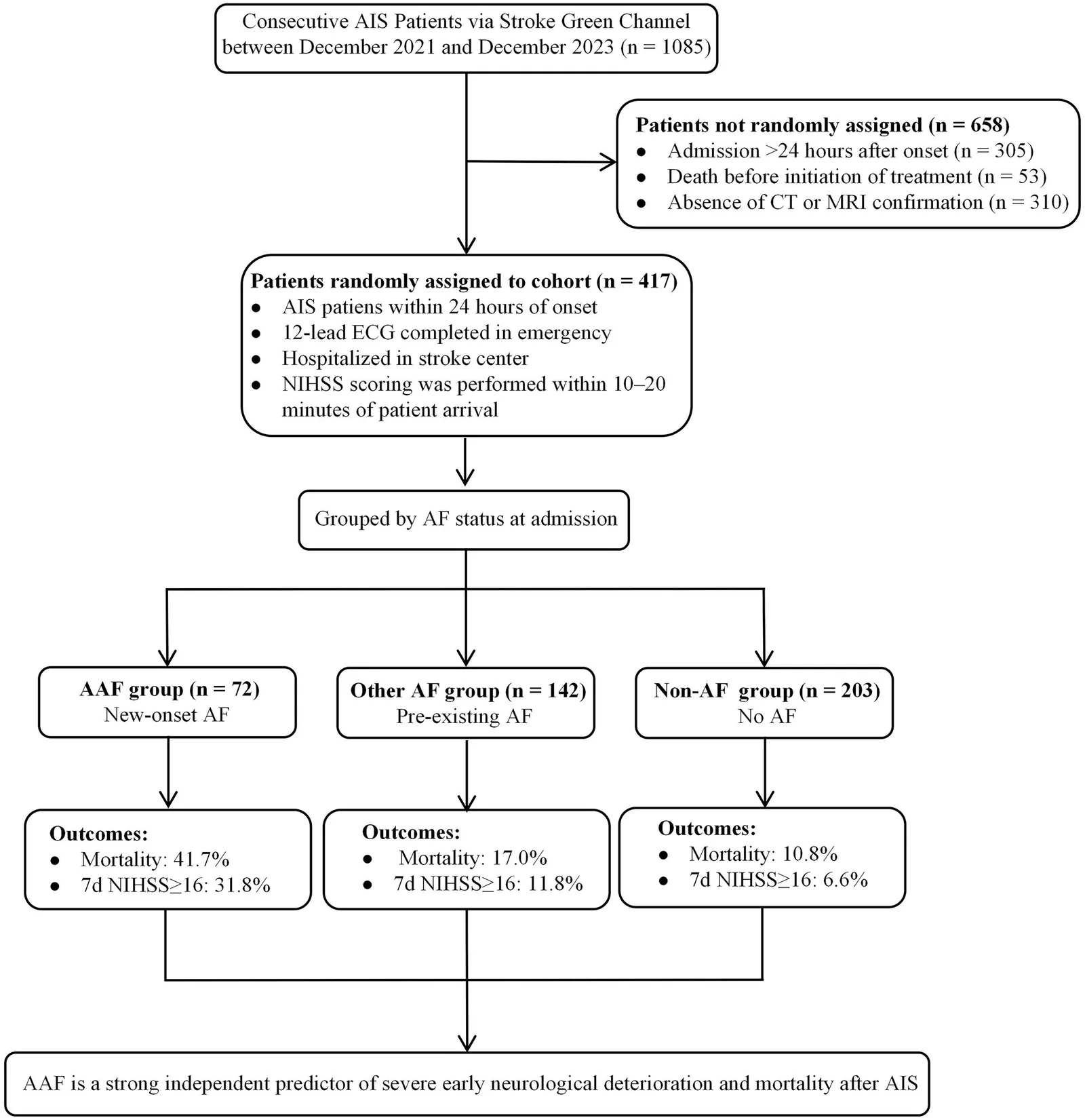
The flowchart.

Patients with AIS admitted to the Sixth People's Hospital Affiliated to Shanghai Jiao Tong University School of Medicine through emergency stroke green channel from December 2021 to December 2023 were retrospectively enrolled in this study. The study protocol was reviewed and approved by the Ethics Committee of Sixth People's Hospital Affiliated to Shanghai Jiao Tong University School of Medicine (2022-KY-111). Written informed consent was obtained from all patients (Figure 1).

### 2.2 Inclusion and exclusion criteria

The inclusion criteria were as follows: (1) diagnosis of AIS within 24 h of onset, (2) completion of a 12-lead electrocardiogram in the emergency room, and (3) admission to the hospital's stroke center.

Exclusion criteria: (1) Admission >24 h after AIS onset; (2) Death before treatment initiation, (3) The results of head computed tomography (CT) or magnetic resonance imaging (MRI) examinations are incomplete, (4) Incomplete clinical or follow-up data, (5) Patients who underwent cardioversion during acute hospitalization.

### 2.3 Diagnostic criteria and patient categorization

AAF was diagnosed by emergency physicians based on 12-lead ECG findings and medical history, following the definition of AF first detected during acute hospitalization or acute illness. The absence of prior AF was determined by patient/family interview and electronic medical record review, confirming no documented AF diagnosis, no AF-related symptoms, and no history of anticoagulant or antiarrhythmic drug use. AIS was diagnosed by neurologists based on permanent focal neurological deficits and confirmed by cranial computed tomography or magnetic resonance imaging.

Patients were classified into three mutually exclusive groups based on their AF status at admission:

AAF Group (*n* = 72): Patients meeting the AAF criteria (newly identified AF during acute stroke presentation, with no prior AF documented in medical records or patient history.).

Other AF Group (*n* = 142): Patients with a documented AF history, verified by the same history-taking and electronic record retrieval process.

Non-AF Group (*n* = 203): Patients with no evidence of AF on index ECG and no history of AF.

### 2.4 Data collection

Demographic information, clinical data, comorbidities, AF-related data, treatment information [intravenous thrombolysis [IT], digital subtraction angiography [DSA]], and National Institutes of Health Stroke Scale (NIHSS) scores at admission and 7 days were extracted from medical records (12).

### 2.5 Outcomes

The primary outcome was all-cause mortality within 30 days. The secondary outcome was the severity of early neurological impairments, characterized by a 7-day NIHSS score of ≥16, which denotes moderate to severe stroke (13, 14).

### 2.6 Data Analysis

Statistical analysis was performed using SPSS 26.0 software, and the significance level was set as α = 0.05. Categorical variables were expressed as frequency (percentage) and compared between groups using Fisher's exact test or chi-square test according to the sample size and theoretical frequency. Kolmogorov–Smirnova and Shapiro–Wilk tests confirmed that continuous variables conformed to the normal distribution and were expressed as mean ± standard deviation, and non-normal distribution was expressed as median (IQR). Multi-group comparisons were conducted using the Kruskal–Wallis H test, followed by *post-hoc* pairwise Mann–Whitney U tests with Bonferroni correction when significant differences were detected. Predictive factors of outcome were determined using univariate and multivariate logistic regression analyses. Variables with *P* < 0.2 in univariate analysis were selected as candidates for entry into the subsequent stepwise multivariate logistic regression model, and a two-tailed *P* < 0.05 was considered statistically significant. The grouping variable (AAF/other AF/non-AF) was forced into all multivariate models as the primary exposure of interest.

## 3 Results

### 3.1 Baseline characteristics

Study participants included 417 consecutive patients with AIS admitted through the emergency stroke green channel and categorized into three groups based on their AF status: AAF (*n* = 72), other AF (pre-existing or other AF) group (*n* = 142) and the non-AF group (*n* = 203). As detailed in Table 1, all three groups were well-balanced at baseline in terms of age, sex, body mass index (BMI), and comorbidities (all *P* > 0.05).

**Table 1:** Baseline and clinical characteristics of the patients with AAF, PAF, or no AF at the time of AIS.

Patient characteristic	AAF(*n* = 72)	Other AF(*n* = 142)	Non AF(*n* = 203)	*P* value
Baseline				
Age (Median, IQR)	83 (73,87)	82.5 (76,87)	81 (76,86)	0.652
Male（%）	29 (40.3)	70 (49.3)	108 (53.2)	0.169
BMI (Median, IQR) kg/m^2^	24.0 (19.7–26.9)	23.3 (20.8–25.9)	23.6 (21.2–25.5)	0.821
HbA1c (Median, IQR) %	5.9 (5.6–6.7)	5.8 (5.5–6.5)	6.1 (5.6–7.2)	0.004
TC (Median, IQR) mmol/L	4.5 (3.8–5.2)	4.2 (3.5–5.1)	4.9 (3.9–5.7)	<0.001
TG (Median, IQR) mmol/L	0.9 (0.7–1.2)	0.8 (0.6–1.1)	1.1 (0.8–1.6)	<0.001
HDL (Median, IQR) mmol/L	1.3 (1.1–1.5)	1.3 (1.0–1.6)	1.2 (1.0–1.4)	0.060
LDL (Median, IQR) mmol/L	2.5 (1.8–3.4)	2.3 (1.7–3.0)	2.9 (2.0–3.6)	<0.001
Baseline NIHSS(Median, IQR)	16.5 (6.5,22)	13 (5,19)	5 (2,12)	<0.001
CAD (%)	11 (15.3)	23 (16.2)	25 (12.3)	0.569
HTN (%)	50 (70.4)	103 (74.6)	143 (70.8)	0.701
CI history (%)	19 (26.4)	38 (26.8)	51 (25.1)	0.938
CH history (%)	2 (2.8)	5 (3.5)	1 (0.5)	0.110
DM history (%)	18 (25.0)	36 (25.4)	76 (37.4)	0.027
**Clinical therapy**				
IT (%)	15 (20.8)	31 (21.8)	56 (27.6)	0.347
DSA (%)	32 (44.4)	59 (41.5)	37 (18.2)	<0.001
Outcome				
7d NIHSS≥16 (%)	23 (31.8)	17 (11.8)	13 (6.6)	<0.001
Mortality (%)	30 (41.7)	24(17.0)	22(10.8)	<0.001

BMI, body mass index; NIHSS, National Institutes of Health Stroke Scale; HbA1c, glycosylated hemo-globin; TC, total cholesterol; TG, triglyceride; HDL, high-density lipoprotein; LDL, low-density lipoprotein; CHA₂DS₂-VASc, congestive heart failure, hypertension, age≥75y (doubled), diabetes mellitus, stroke (dou-bled)-vascular disease, age 65–74 and sex category (female); CAD, Coronary Heart Disease; HTN, hypertension; CI history, history of cerebral infarction; CH history, history of cerebral hemorrhage; DM history, history of diabetes; IT, intravenous thrombolysis; DSA, digital subtraction angiography.

Data were shown as mean ± SD, frequency, and percentage. SD, standard deviation; *P < 0.05 vs. Control group.

Upon admission, the AAF cohort exhibited the most pronounced neurological impairments, as indicated by a substantially elevated median baseline NIHSS score compared with the other AF and non-AF groups (16.5 vs. 13 vs. 5, *P* < 0.001). The AAF group had a more favorable lipid profile, with significantly lower TC, TG, and LDL levels (all *P* < 0.001). The HbA1c level was also lower in the AAF group than in the non-AF group (*P* = 0.004). Regarding clinical management, the proportion of patients with other AF was significantly higher than that of patients with AAF and non-AF (*P* < 0.001).

Outcome measures demonstrated a substantially higher burden of adverse events in the AAF group than in the other two groups. The 30-day all-cause mortality rate was 41.7% (30/72) in the AAF group, significantly exceeding that of the other AF (16.9%, 24/142) and non-AF (10.8%, 22/203) groups (*P* < 0.001) (Table 1). Similarly, the incidence of severe neurological deficit at 7 days (defined as NIHSS ≥ 16) was significantly higher in the AAF group (31.8%, 23/72) than in the other AF (12.0%, 17/142) and non-AF (6.4%, 13/203) groups (*P* < 0.001) (Table 1).

### 3.2 Regression analysis of the 30-day mortality

Univariate and multivariate logistic regression analyses were used to assess whether AAF at 30 days was an independent risk factor for death (Table 2). In the univariate analysis, AAF was strongly associated with the risk of death compared with the non-AF group (death (OR = 6.24, 95% CI: 3.37–11.68, *P* < 0.001) (Table 2).

**Table 2:** Univariate and multivariate logistic regression analysis of 30-day mortality.

	Univariate analysis		Multivariate analysis			
			Model 1		Model 2	
	OR(95%CI)	*P*	OR (95%CI)	*P*	OR (95%CI)	*P*
Age						
< 65	REF		REF			
65–73	0.15 (0.02, 1.31)	0.066				
73–84	0.15 (0.02, 1.28)	0.062				
>=84	0.31 (0.04, 2.63)	0.247	1.30 (0.12, 31.26)	0.839		
Sex-Female	1.63 (1.01, 2.67)	0.049	0.91 (0.41, 2.02)	0.806		
BMI	1.01 (0.96, 1.07)	0.632				
HbA1c	1.00 (0.83, 1.18)	0.997				
TC	0.96 (0.78, 1.17)	0.693				
TG	0.58 (0.34, 0.91)	0.028	0.35 (0.15, 0.80)	0.017		
HDL	1.86 (0.95, 3.59)	0.065	1.41 (0.51, 3.97)	0.507		
LDL	0.91 (0.70, 1.17)	0.462				
Baseline NIHSS	1.12 (1.09, 1.15)	<0.001	1.11 (1.07, 1.16)	<0.001	1.13 (1.10, 1.16)	<0.001
Group						
Non AF	REF		REF			
Other AF	1.79 (0.98, 3.26)	0.056	0.34 (0.13, 0.84)	0.022	1.08 (0.64, 1.81)	0.778
AAF	6.24 (3.37, 11.68)	<0.001	2.04 (0.80, 5.19)	0.132	4.11 (2.28, 7.43)	<0.001
CAD	1.33 (0.66, 2.53)	0.395				
HTN	1.58 (0.90, 2.95)	0.128	7.77 (2.99, 23.59)	<0.001	3.59 (1.90, 7.24)	<0.001
CI history	1.13 (0.65, 1.92)	0.658				
CH history	1.39 (0.20, 5.87)	0.686				
DM history	0.87 (0.51, 1.47)	0.616				

In the multivariate analysis, two models with different adjustment levels were constructed. In Model 1 (adjusted for TG, HDL, baseline NIHSS, AF group, and HTN), the association between AAF and mortality was attenuated and loss of statistical significance was observed (OR = 2.04, 95% CI: 0.80–5.19, *P* = 0.132) (Table 2). However, in Model 2 (a more parsimonious model adjusted for the potent confounders baseline NIHSS, AF group, and HTN), AAF was again a strong independent predictor of mortality. Compared with patients without atrial fibrillation, the probability of death within 30 days for AAF patients increases by 4.11 times (OR = 4.11, 95% CI: 2.28–7.43, *P* < 0.001) (Table 2).

### 3.3 Regression Analysis for 7-Day Severe Neurological Deficit

The analysis of severe neurological deficit at 7 days further underscored the negative impact of AAF (Table 3). Univariate analysis indicated that patients with AAF had an 8.44-fold higher risk of severe neurological deficit compared with the non-AF group (OR = 8.44, 95% CI: 3.58–20.29, *P* < 0.001) (Table 3).

**Table 3:** Univariate and multivariate logistic regression analysis of 7-day adverse outcomes.

	Univariate analysis		Multivariate analysis			
			Model 1		Model 2	
	OR(95%CI)	*P*	OR (95%CI)	*P*	OR (95%CI)	*P*
Age						
< 65	REF		REF			
65–73	0.01 (0.00,0.17)	0.002				
73–84	0.05 (0.00,0.54)	0.016				
>=84	0.09 (0.00,1.01)	0.057	0.14 (0.00,2.51)	0.203		
Sex-Female	1.74 (0.90,3.42)	0.101	3.14 (0.92,12.38)	0.080		
BMI	1.02 (0.94,1.09)	0.652				
HbA1c	1.00 (0.77,1.23)	0.976				
TC	1.04 (0.80,1.34)	0.759				
TG	0.90 (0.52,1.39)	0.671	1.85 (0.72,4.20)	0.165		
HDL	0.80 (0.29,2.06)	0.653	0.44 (0.08,2.22)	0.332		
LDL	1.08 (0.77,1.49)	0.636				
Baseline NIHSS	1.18 (1.13,1.24)	<0.001	1.22 (1.14,1.32)	<0.001	1.19 (1.14,1.25)	<0.001
Group						
Non AF	REF		REF			
Other AF	2.41 (1.08,5.48)	0.032	1.25 (0.33,4.80)	0.745	1.31 (0.56,3.12)	0.541
AAF	8.44 (3.58,20.29)	<0.001	2.66 (0.64,10.90)	0.171	10.09 (3.87,27.36)	<0.001
CAD	1.27 (0.46,3.00)	0.615				
HTN	0.72 (0.37,1.48)	0.358	0.69 (0.23,2.09)	0.496	1.31 (0.58,3.16)	0.528
CI history	0.88 (0.38,1.86)	0.758				
CH history	7.05 (1.35,33.19)	0.013				
DM history	1.06(0.51,2.09)	0.872				

Similar patterns of observed and mortality were analyzed using multivariate models. After adjustment for several variables in Model 1, the association between AAF and severe neurological deficit was significant (OR = 2.66, 95% CI: 0.64–10.90, *P* = 0.171). Conversely, in Model 2 (adjusted for baseline NIHSS, AF group, and HTN), AAF was robustly and independently associated with adverse neurological outcomes, increasing the probability of severe neurological impairment by more than 10 times (OR = 10.09, 95% CI: 3.87–27.36, *P* < 0.001) (Table 3).

## 4 Discussions

The key result of this investigation indicates that AAF serves as a robust and independent prognostic marker for unfavorable short-term outcomes in AIS. Specifically, our multivariate analyses demonstrate that AAF significantly increases the risk of severe neurological deficits at 7 days and higher 30-day all-cause mortality. These results firmly establish AAF not only as an incidental comorbidity but as a critical risk factor that profoundly influences the early clinical course after AIS.

Although previous studies have shown that patients with AIS and pre-existing AF present with greater neurological impairment and face worse prognoses (15–17), our research specifically highlights the heightened vulnerability associated with the new-onset and acute presentation of the arrhythmia in this setting. The strikingly high prevalence of severe 7-day neurological deficits (31.8%) in our AAF cohort, coupled with an adjusted odds ratio of 10.09 in our primary model, underscores that AF onset acuity is a crucial prognostic determinant. This suggests a potential synergistic effect between the new, unstable cardiac rhythm and the acute cerebral insult, leading to more extensive brain injury or hampering early recovery.

The pathophysiological relationship between AIS and AF is complex and bidirectional (18, 19). On the one hand, the direct cause of stroke in AF may be cardiac emboli originating from the newly dysfunctional atria (20). On the other hand, the acute cerebral infarction itself can act as a potent trigger for AF through neurogenic mechanisms, including dysregulation of the autonomic nervous system and a surge in catecholamines (21). This “stroke-induced” AAF may then exacerbate the initial injury by promoting a pro-thrombotic and pro-inflammatory state, potentially leading to clot propagation, impaired cerebral perfusion, and increased mortality, as showed in our 30-day data. The poor prognosis associated with AAF may be explained by the following mechanisms: the rapid ventricular response in AAF compromises cardiac output and cerebral perfusion, while loss of atrial contraction promotes left atrial appendage stasis and thrombogenesis. Concurrent systemic inflammation and hypercoagulability, coupled with absent pre-stroke anticoagulation, further amplify thrombotic risk and ischemic burden. Our observation that AAF remained an independent predictor of mortality (OR = 4.11) after adjusting for baseline stroke severity reinforces the concept that its presence adds a significant and distinct layer of risk beyond the initial infarct.

The identification of AAF concurrent with AIS is a paramount indicator of high risk for early neurological deterioration and death, demanding a more aggressive and vigilant management strategy. Requiring monitoring in a stroke unit or step-down setting is warranted to detect and manage potential complications promptly. Furthermore, the diagnosis of AAF urgently raises the urgency of considering anticoagulation for secondary stroke prevention. While the optimal timing for initiating anticoagulation after an AIS remains a nuanced decision, our data emphasize AAF as a central factor in this risk-benefit calculus. Early cardiology consultation should be considered to guide rhythm or rate control and to inform long-term anti-coagulation strategies.

There are several limitations to this study. First, the single-center and retrospective design might have limited the general applicability of our research results. Second, although sufficient for the primary analysis, the sample size of the AAF subgroup have influenced the stability of the multivariate model for mortality across different covariate adjustments. Third, the retrospective nature of data collection carries an inherent risk of incomplete documentation regarding the precise duration and management of AAF episodes. Fourth, the diagnosis of AAF and Non-AF relied on medical records and routine ECG rather than prospective continuous rhythm monitoring, which may present a classification limitation. Finally, our study focused on short-term outcomes. Future prospective studies are needed to investigate AAF on functional recovery, as well as the long-term effects of AF recurrence of load stroke.

## 5 Conclusions

This investigation yields compelling evidence that AAF constitutes a potent autonomous risk factor for marked early neurological worsening and fatality after AIS. Detection of new-onset AF during an acute stroke is not a benign finding but a pivotal prognostic marker that should serve as an impetus for enhanced monitoring and inform critical early therapeutic decisions. Integrating AAF status into initial risk stratification models could significantly improve the early identification and management of the patients with AIS.

## Data Availability

The original contributions presented in the study are included in the article/Supplementary Material, further inquiries can be directed to the corresponding author.
