# Supplementary material for: Predictive value of acute atrial fibrillation for short-term mortality after ischemic stroke
Source: Front Cardiovasc Med. 2026 Aug 13;13:1828038. doi: 10.3389/fcvm.2026.1828038 (PMC13519392; doi:10.3389/fcvm.2026.1828038)
Supplement: Supplementary file 1 [file Supplementaryfile1.docx]

STROBE Statement—checklist of items that should be included in reports of observational studies

|  | Item No. | Recommendation | Page  No. | Relevant text from manuscript |
| --- | --- | --- | --- | --- |
| **Title and abstract** | 1 | (*a*) Indicate the study’s design with a commonly used term in the title or the abstract | Abstract | This study categorized 417 patients with AIS into the AAF (n = 72), pre-existing AF (n = 142), and non-AF (n = 203) groups. |
|  |  | (*b*) Provide in the abstract an informative and balanced summary of what was done and what was found | Abstract | The AAF group demonstrated significantly worse outcomes than the other AF and non-AF groups. In the multivariable analysis, AAF was identified as an independent risk predictor for severe 7-day neurological deficit (odds ratio [OR]: 10.09; 95% CI: 3.87-27.36; P < 0.001) and all-cause mortality within 30 days (OR: 4.11; 95% CI: 2.28-7.43; P < 0.001). |
| Introduction | | | |  |
| Background/rationale | 2 | Explain the scientific background and rationale for the investigation being reported | Introduction | Atrial fibrillation (AF) accounts for about 2–4% of adults, and its incidence has gradually increased in recent years. AF is the most common arrhythmia, and it is a key risk factor in the emergency room with acute ischemic stroke. Approximately 15% of strokes are linked to AF, and ischemic strokes associated with AF tend to be more debilitating and have a higher mortality risk than other subtypes.  Acute atrial fibrillation (AAF) is defined by the American Heart Association as AF detected or manifested during an emergency department admission. Patients with AAF during AIS presentation were distinguished from those with pre-existing AF, representing 25%–36.2% of patients after their first stroke. Patients with AAF, particularly following a cerebrovascular event, are at risk of recurrence, stroke, and poor outcomes. Consequently, recognizing the impact of AAF on AIS outcomes is key to therapeutic decision-making, including the timing of cardiac monitoring and anticoagulation to prevent stroke recurrence |
| Objectives | 3 | State specific objectives, including any prespecified hypotheses | Introduction | This study aimed to assess the predictive value of AAF for early neurological deterioration and short-term mortality in AIS patients. |
| Methods | | | |  |
| Study design | 4 | Present key elements of study design early in the paper | Methods | Patients with AIS admitted to the Sixth People’s Hospital Affiliated to Shanghai Jiao Tong University School of Medicine through emergency stroke green channel from December 2021 to December 2023 were retrospectively enrolled in this study. |
| Setting | 5 | Describe the setting, locations, and relevant dates, including periods of recruitment, exposure, follow-up, and data collection | Methods |  |
| Participants | 6 | (*a*) *Cohort study*—Give the eligibility criteria, and the sources and methods of selection of participants. Describe methods of follow-up  *Case-control study*—Give the eligibility criteria, and the sources and methods of case ascertainment and control selection. Give the rationale for the choice of cases and controls  *Cross-sectional study*—Give the eligibility criteria, and the sources and methods of selection of participants | Methods | The inclusion criteria were as follows: (1) diagnosis of AIS within 24 h of onset, (2) completion of a 12-lead electrocardiogram in the emergency room, and (3) admission to the hospital’s stroke center.  Exclusion criteria: (1) Admission >24 hours after AIS onset; (2) Death before treatment initiation, (3) The results of head computed tomography (CT) or magnetic resonance imaging (MRI) examinations are incomplete, (4) Incomplete clinical or follow-up data. |
|  |  | (*b*) *Cohort study*—For matched studies, give matching criteria and number of exposed and unexposed  *Case-control study*—For matched studies, give matching criteria and the number of controls per case | **Not applicable** |  |
| Variables | 7 | Clearly define all outcomes, exposures, predictors, potential confounders, and effect modifiers. Give diagnostic criteria, if applicable | Methods | The primary outcome was all-cause mortality within 30 days. The secondary outcome was the severity of early neurological impairments, characterized by a 7-day NIHSS score of ≥16, which denotes moderate to severe stroke. |
| Data sources/ measurement | 8* | For each variable of interest, give sources of data and details of methods of assessment (measurement). Describe comparability of assessment methods if there is more than one group | Methods | Demographic information, clinical data, comorbidities, AF-related data, treatment information (intravenous thrombolysis [IT], digital subtraction angiography [DSA]), and National Institutes of Health Stroke Scale (NIHSS) scores at admission and 7 days were extracted from medical records. |
| Bias | 9 | Describe any efforts to address potential sources of bias | **Not applicable** |  |
| Study size | 10 | Explain how the study size was arrived at | **Not applicable** |  |

Continued on next page

| Quantitative variables | 11 | Explain how quantitative variables were handled in the analyses. If applicable, describe which groupings were chosen and why | Methods | Kolmogorov-Smirnova and Shapiro-Wilk tests confirmed that continuous variables conformed to the normal distribution and were expressed as mean ± standard deviation, and non-normal distribution was expressed as median (IQR). |
| --- | --- | --- | --- | --- |
| Statistical methods | 12 | (*a*) Describe all statistical methods, including those used to control for confounding | Methods | Predictive factors of outcome were determined using univariate and multivariate logistic regression analyses. |
|  |  | (*b*) Describe any methods used to examine subgroups and interactions | **Not applicable** |  |
|  |  | (*c*) Explain how missing data were addressed | **Not applicable** |  |
|  |  | (*d*) *Cohort study*—If applicable, explain how loss to follow-up was addressed  *Case-control study*—If applicable, explain how matching of cases and controls was addressed  *Cross-sectional study*—If applicable, describe analytical methods taking account of sampling strategy | Methods | Exclusion criteria: (1) Admission >24 hours after AIS onset; (2) Death before treatment initiation, (3) The results of head computed tomography (CT) or magnetic resonance imaging (MRI) examinations are incomplete, (4) Incomplete clinical or follow-up data. |
|  |  | (*e*) Describe any sensitivity analyses | **Not applicable** |  |
| Results | | | | |
| Participants | 13* | (a) Report numbers of individuals at each stage of study—eg numbers potentially eligible, examined for eligibility, confirmed eligible, included in the study, completing follow-up, and analysed | Results | Study participants included 417 consecutive patients with AIS admitted through the emergency stroke green channel and categorized into three groups based on their AF status: AAF (n = 72), pre-existing AF (pre-existing or pre-existing AF) group (n = 142) and the non-AF group (n = 203). |
|  |  | (b) Give reasons for non-participation at each stage | **Not applicable** |  |
|  |  | (c) Consider use of a flow diagram | Figure Legend | Figure1 |
| Descriptive data | 14* | (a) Give characteristics of study participants (eg demographic, clinical, social) and information on exposures and potential confounders | Results | As detailed in **Table 1**, all three groups were well-balanced at baseline in terms of age, sex, body mass index (BMI), and comorbidities (all *P* > 0.05). |
|  |  | (b) Indicate number of participants with missing data for each variable of interest | **Not applicable** |  |
|  |  | (c) *Cohort study*—Summarise follow-up time (eg, average and total amount) | Results | The 30-day all-cause mortality rate was 41.7% in the AAF group, significantly exceeding that of the pre-existing AF (17.0%) and non-AF (10.8%) groups (*P* < 0.001) (**Table 1**). Similarly, the incidence of severe neurological deficit at 7 days (defined as NIHSS ≥ 16) was significantly higher in the AAF group (31.8%) than in the pre-existing AF (11.8%) and non-AF (6.6%) groups (*P* < 0.001) |
| Outcome data | 15* | *Cohort study*—Report numbers of outcome events or summary measures over time | Results | The 30-day all-cause mortality rate was 41.7% in the AAF group, significantly exceeding that of the pre-existing AF (17.0%) and non-AF (10.8%) groups (P < 0.001) (**Table 1**). Similarly, the incidence of severe neurological deficit at 7 days (defined as NIHSS ≥ 16) was significantly higher in the AAF group (31.8%) than in the pre-existing AF (11.8%) and non-AF (6.6%) groups (P < 0.001). |
|  |  | *Case-control study—*Report numbers in each exposure category, or summary measures of exposure |  |  |
|  |  | *Cross-sectional study—*Report numbers of outcome events or summary measures |  |  |
| Main results | 16 | (*a*) Give unadjusted estimates and, if applicable, confounder-adjusted estimates and their precision (eg, 95% confidence interval). Make clear which confounders were adjusted for and why they were included | Results | In the univariate analysis, AAF was strongly associated with the risk of death compared with the non-AF group (death (OR = 6.24, 95% CI: 3.37–11.68, *P* < 0.001) (Table 2).  Compared with patients without atrial fibrillation, the probability of death within 30 days for AAF patients increases by 4.11 times (OR = 4.11, 95% CI: 2.28–7.43, *P* < 0.001) (Table 2).  Univariate analysis indicated that patients with AAF had an 8.44-fold higher risk of severe neurological deficit compared with the non-AF group (OR = 8.44, 95% CI: 3.58–20.29, *P* < 0.001) (Table 3).  AAF was robustly and independently associated with adverse neurological outcomes, increasing the probability of severe neurological impairment by more than 10 times (OR = 10.09, 95% CI: 3.87–27.36, *P* < 0.001) (Table 3). |
|  |  | (*b*) Report category boundaries when continuous variables were categorized | Results | Similarly, the incidence of severe neurological deficit at 7 days (defined as NIHSS ≥ 16) was significantly higher in the AAF group (31.8%) than in the pre-existing AF (11.8%) and non-AF (6.6%) groups (*P* < 0.001) (Table 1). |
|  |  | (*c*) If relevant, consider translating estimates of relative risk into absolute risk for a meaningful time period | Results | The 30-day all-cause mortality rate was 41.7% in the AAF group, significantly exceeding that of the pre-existing AF (17.0%) and non-AF (10.8%) groups (*P* < 0.001) (Table 1). |

Continued on next page

| Other analyses | 17 | Report other analyses done—eg analyses of subgroups and interactions, and sensitivity analyses | Results | In the multivariate analysis, two models with different adjustment levels were constructed. In Model 1 (adjusted for TG, HDL, baseline NIHSS, AF group, and HTN), the association between AAF and mortality was attenuated and loss of statistical significance was observed (OR = 2.04, 95% CI: 0.80–5.19, *P* = 0.132) (Table 2). However, in Model 2 (a more parsimonious model adjusted for the potent confounders baseline NIHSS, AF group, and HTN), AAF was again a strong independent predictor of mortality. Compared with patients without atrial fibrillation, the probability of death within 30 days for AAF patients increases by 4.11 times (OR = 4.11, 95% CI: 2.28–7.43, *P* < 0.001) (Table 2). |
| --- | --- | --- | --- | --- |
| Discussion | | | | |
| Key results | 18 | Summarise key results with reference to study objectives | Discussion | The key result of this investigation indicates that AAF serves as a robust and independent prognostic marker for unfavorable short-term outcomes in AIS. Specifically, our multivariate analyses demonstrate that AAF significantly increases the risk of severe neurological deficits at 7 days and higher 30-day all-cause mortality. These results firmly establish AAF not only as an incidental comorbidity but as a critical risk factor that profoundly influences the early clinical course after AIS. |
| Limitations | 19 | Discuss limitations of the study, taking into account sources of potential bias or imprecision. Discuss both direction and magnitude of any potential bias | Discussion | There are several limitations to this study. First, the single-center and retrospective design might have limited the general applicability of our research results. Second, although sufficient for the primary analysis, the sample size of the AAF subgroup have influenced the stability of the multivariate model for mortality across different covariate adjustments. Third, the retrospective nature of data collection carries an inherent risk of incomplete documentation regarding the precise duration and management of AAF episodes. Finally, our study focused on short-term outcomes. Future prospective studies are needed to investigate AAF on functional recovery, as well as the long-term effects of AF recurrence of load stroke. |
| Interpretation | 20 | Give a cautious overall interpretation of results considering objectives, limitations, multiplicity of analyses, results from similar studies, and other relevant evidence | Discussion | The identification of AAF concurrent with AIS is a paramount indicator of high risk for early neurological deterioration and death, demanding a more aggressive and vigilant management strategy. Requiring monitoring in a stroke unit or step-down setting is warranted to detect and manage potential complications promptly. Furthermore, the diagnosis of AAF urgently raises the urgency of considering anticoagulation for secondary stroke prevention. While the optimal timing for initiating anticoagulation after an AIS remains a nuanced decision, our data emphasize AAF as a central factor in this risk-benefit calculus. Early cardiology consultation should be considered to guide rhythm or rate control and to inform long-term anti-coagulation strategies. |
| Generalisability | 21 | Discuss the generalisability (external validity) of the study results | Discussion | First, the single-center and retrospective design might have limited the general applicability of our research results. Second, although sufficient for the primary analysis, the sample size of the AAF subgroup have influenced the stability of the multivariate model for mortality across different covariate adjustments. Third, the retrospective nature of data collection carries an inherent risk of incomplete documentation regarding the precise duration and management of AAF episodes. Finally, our study focused on short-term outcomes. Future prospective studies are needed to investigate AAF on functional recovery, as well as the long-term effects of AF recurrence of load stroke. |
| Other information | |  | | |
| Funding | 22 | Give the source of funding and the role of the funders for the present study and, if applicable, for the original study on which the present article is based | Statement | This work was supported by Clinical Research Funds of Sixth People’s Hospital Affiliated to Shanghai Jiao Tong University School of Medicine (Project No. ynhg202208 and ynhg202311). |

*Give information separately for cases and controls in case-control studies and, if applicable, for exposed and unexposed groups in cohort and cross-sectional studies.

**Note:** An Explanation and Elaboration article discusses each checklist item and gives methodological background and published examples of transparent reporting. The STROBE checklist is best used in conjunction with this article (freely available on the Web sites of PLoS Medicine at http://www.plosmedicine.org/, Annals of Internal Medicine at http://www.annals.org/, and Epidemiology at http://www.epidem.com/). Information on the STROBE Initiative is available at www.strobe-statement.org.
